# Analysis of the microstates of prolonged disorders of consciousness

**DOI:** 10.3389/fneur.2025.1659809

**Published:** 2025-10-29

**Authors:** Yuzhang Wu, Shangang Feng, Deqiu Cui, Zhongzhen Li, Ruowei Qu, Yifeng Cheng, Yangang Wang, Shaoya Yin, Keke Feng

**Affiliations:** ^1^Department of Neurosurgery, Tianjin Huanhu Hospital, Tianjin, China; ^2^Huanhu Hospital Affiliated to Tianjin Medical University, Tianjin, China; ^3^State Key Laboratory of Reliability and Intelligence of Electrical Equipment, Hebei University of Technology, Tianjin, China; ^4^Department of Neurosurgery, The People’s Hospital of Jiaozuo City, Jiaozuo, Henan, China

**Keywords:** prolonged disorders of consciousness, EEG, microstate, brain connectivity, global field power

## Abstract

**Objectives:**

Prolonged disorders of consciousness (pDOC) is a common disease in neurology. This study aimed to explore the microstate (MS) characteristics of pDOC patients based on resting-state Electroencephalography (EEG) and explore the changes in brain function in pDOC patients.

**Methods:**

Patients were divided into healthy group and pDOC group according to the presence of consciousness. The T1 weighted 3D magnetization pre-gradient echo sequence (3d-t1-mprage) and resting state video EEG data were collected. First, the microstate clustering analysis was carried out. The global field power (GFP) was used to calculate microstate indicators (Duration, Occurrence, and Coverage), and then the statistical analysis between groups was carried out. Finally, the original resting state EEG data were reconstructed according to the clustered microstate GFP peak, and the connectivity of the brain in the four microstates was calculated, respectively.

**Results:**

The topographic map of MS B in the DOC goup (DG) is different from that in the healthy goup (HG). The Coverage value of MS B (*Z* = −2.084, *p* = 0.037), the Occurrence value of MS D (*Z* = −2.141, *p* = 0.032), the Coverage value of MS D (*Z* = −1.999, *p* = 0.046) between the two groups has a significant difference. The MS B topographic map was significantly different between the two groups. The MS D of DG was significantly different from that of the HG. Only part of the connections were preserved.

**Conclusion:**

The microstate topographic maps of pDOC and CG are not identical. At the same time, the brain connectivity of pDOC patients in the four MS decreased significantly compared to the HG.

## Introduction

Prolonged disorder of consciousness (pDOC) refers to the conditions characterized by prolonged loss of awareness for more than 28 days (1). pDOC is divided into vegetative state (VS) and minimal conscious state (MCS). VS is also known as unresponsive wakefulness syndrome (UWS), which refers to the presence of basic brainstem reflexes and sleep-waking cycles, without content of consciousness (2). MCS refers to discontinuous and fluctuating signs of consciousness in patients (3). In recent years, with the progress of medical technology, it has been found that some pDOC patients show signs of brain activity, indicating that their brain has hidden consciousness. Therefore, some new diagnostic classifications have been proposed, such as “Cognitive motor dissociation,” “Recessive cortical activity,” and “MCS*” (4–6). The “mescocircurt mode “proposed in recent years believes that the connections of the thalamus, frontal lobe, parietal, occipital, and temporal sensory cortex are the basic circuits of consciousness, and the damage of changing the circuit will lead to the disorders of consciousness (7). However, questions about where consciousness comes from and what factors are related to the level of consciousness are still being explored.

Research has confirmed that the brain is not inactive when there is no stimulation. On the contrary, the brain will be active in an organized way at rest, to prepare for the next possible stimulation processing (8). Functional magnetic resonance imaging (fMRI) is one of the tools to study brain function. fMRI relies on the difference of magnetization vector between oxyhemoglobin and deoxyhemoglobin to generate the signal, which indirectly displays the activity of brain tissue with blood oxygenation level dependent (BOLD) signal (9). fMRI can generate a statistical map of the whole brain connections of a specific region or network by associating the BOLD signal in the region of interest (ROI) with all other voxels in the brain (10). Moreover, fMRI can map brain network components closely related to consciousness, such as default mode network (DMN), salience network (SN), and executive control network (ECN), and serve as neuroimaging biomarkers for pDOC prognosis (11, 12). However, the neurons are constantly active, and the time resolution of fMRI cannot meet the requirements of monitoring the complete sequence of brain activity.

Electroencephalography (EEG) is a noninvasive examination. The principle of EEG is that the image obtained by amplifying and recording the spontaneous biological potential of the brain from the scalp is the spontaneous and rhythmic electrical activity of brain cell groups recorded by electrodes. For pDOC patients, resting-state EEG has become the main modality in electrophysiological evaluation in clinic work. One of the advantages of EEG is its high temporal resolution, which is more suitable for studying the time series of resting state. EEG can be used to assess the integrity of patients’ sleep-waking cycle (13), and studies have found that the presence of sleep spindles is associated with consciousness (14). pDOC patients do not have systemic changes in the sleep spindle and slow wave oscillations between day and night (15). Event-related potential (ERP) is a kind of special evoked potential, which refers to the bioelectric response that can be detected in the system and the corresponding parts of the brain, and has a time-locked relationship with the stimulus and a specific location phase when given a specific stimulus to the brain, or the brain processes the stimulus information. The ERP is based on EEG. At present, P300 and mismatch negativity (MMN) are the most widely used. P300 is a positive wave that appears about 300 ms after stimulation triggered by the Oddball paradigm. It can be found in healthy people, MCS, and Locked-in syndrome patients, but it is rare in UWS (16). P300 reflects the brain’s ability to process information. The amplitude of P300 is correlated with the Coma Recovery Scale-Revise (CRS-R) score, and P300 latency is prolonged in pDOC patients (17–19). MMN is to superimpose and average the ERP of standard stimuli and deviant stimuli respectively, and subtract the ERP of standard stimuli from the ERP of deviant stimuli to obtain the difference wave. The negative deflection of 100-250 ms after the stimulus difference is MMN (20). MMN can distinguish healthy patients from doc patients, and the amplitude of MMN is often lower in UWS (21, 22). EEG spectral power, functional connectivity, graph theory, and nonlinear measurement are also widely used in the study of pDOC (16). Among various brain connectivity measurement methods, EEG-based brain connectivity uniquely provides resolution on the millisecond scale. Similar to fMRI, EEG can be used to construct a multiscale brain network consisting of nodes (hubs) and connections between them, in which the topology can be quantified. Some studies have proved the use of complex network analysis for pDOC by extracting the EEG parameter of network topology in the resting state and can distinguish MCS from VS (23, 24). pDOC patients have reduced local and overall efficiency of the resting state network and fewer hubs in the alpha band (25). Quantitative electroencephalogram (QEEG) is based on the classical EEG that records the activities of brain neurons. It converts the original, complex, and changeable electrophysiological curve into quantitative, and orderly data, thereby improving the sensitivity to changes in brain function (26). At present, many EEG indicators with prognostic potential have been found, such as (*δ* + *θ*)/ (*α* + *β*) Ratio (DTABR), etc., can predict the prognosis of pDOC (27).

In 1987, Lehmann et al. showed that the alpha frequency band (8-12 Hz) of resting-state EEG signals can be resolved into a limited number of distinct quasi-stable states (28). These discrete states are referred to as “microstates (MS)” and are calculated to remain stable for 80–120 ms before rapidly transitioning to other MS (28). In most normal EEG microstate studies, the four microstate maps obtained by clustering have high similarity (29). Specifically, MS 1 shows an upper left and lower right direction, MS 2 shows an upper right and lower left direction, MS 3 shows a forward-backward direction, and MS 4 shows a midline frontaltopographies, the maximum values distributed in frontal and central parts. Even if more clustering graphs are selected, these four MS always dominate in different age ranges, conditions (such as sleep and hypnosis), and pathological states. MS analysis considers signals from all electrodes on the scalp to display functional status globally, rather than individual electrode signals. The rich time series of MS provides a new quantification of EEG signals. Many studies have shown that the characteristics of MS time series of EEG vary with different behavioral states (30). MS analysis is a method to study the neural characteristics of many cognitive processes, and also a method to study brain dynamic functions and link these dynamics with cognition and disease. MS dynamics are closely related to perceptual consciousness, vision, neuropsychiatric disorders, including schizophrenia, resting-state functional network, etc. (31). Lehmann et al. believe that various MS extracted from the brain’s electric field have different levels of consciousness and psychological support, and MS are considered “atoms of thought” (32). Current research indicates that MS is associated with various mental and neurological diseases. Schizophrenia is a chronic disease with unknown etiology, usually manifested as a syndrome with varying symptoms, involving various disorders such as sensory perception, thinking, emotion, and behavior, as well as uncoordinated mental activities. In 2016, Rieger et al. conducted a meta-analysis showing the medium-sized effects of MS 3 and MS 4. The Occurrence of MS 3 is more frequent in patients with schizophrenia, while MS 4 had a shorter Duration, and MS 2 also had a shorter Duration, although not significantly (33). MS 3 and MS 4 are considered early markers of the risk of schizophrenia development, and drug treatment can normalize the patterns of these two MS. Therefore, MS can not only predict the development of certain diseases but also monitor treatment outcomes. Ricci et al. found that after 3 months of treatment with levetiracetam for temporal lobe epilepsy, the direction of MS maps and related indicators in epileptic patients changed, and proposed that MS can be used as a biomarker for the efficacy of levetiracetam in treating epilepsy (34). Similarly, MS are considered markers of diseases such as stroke, severe depression, migraine, and Parkinson’s disease (35–38). It can also study the physiological state of the brain. A study shows that MS 3 and 4 play a dominant role during Non-rapid eye movement sleep, and explains why the dreaming brain will disconnect the dreaming process and keep sleeping (39).

There are significant differences in brain function between pDOC patients and healthy individuals. Current research has focused on P300, BOLD, and other studies (40), but there is relatively little research on the MS of pDOC. EEG has the advantages of relatively low price, bedside monitoring, and easy to carry out in basic hospitals. This paper uses the MS analysis based on EEG, combined with brain connectivity analysis, to explore the differences between pDOC and normal brain, and to explore appropriate biomarkers in clinical practice.

## Method

### Participants

**Figure 1:**
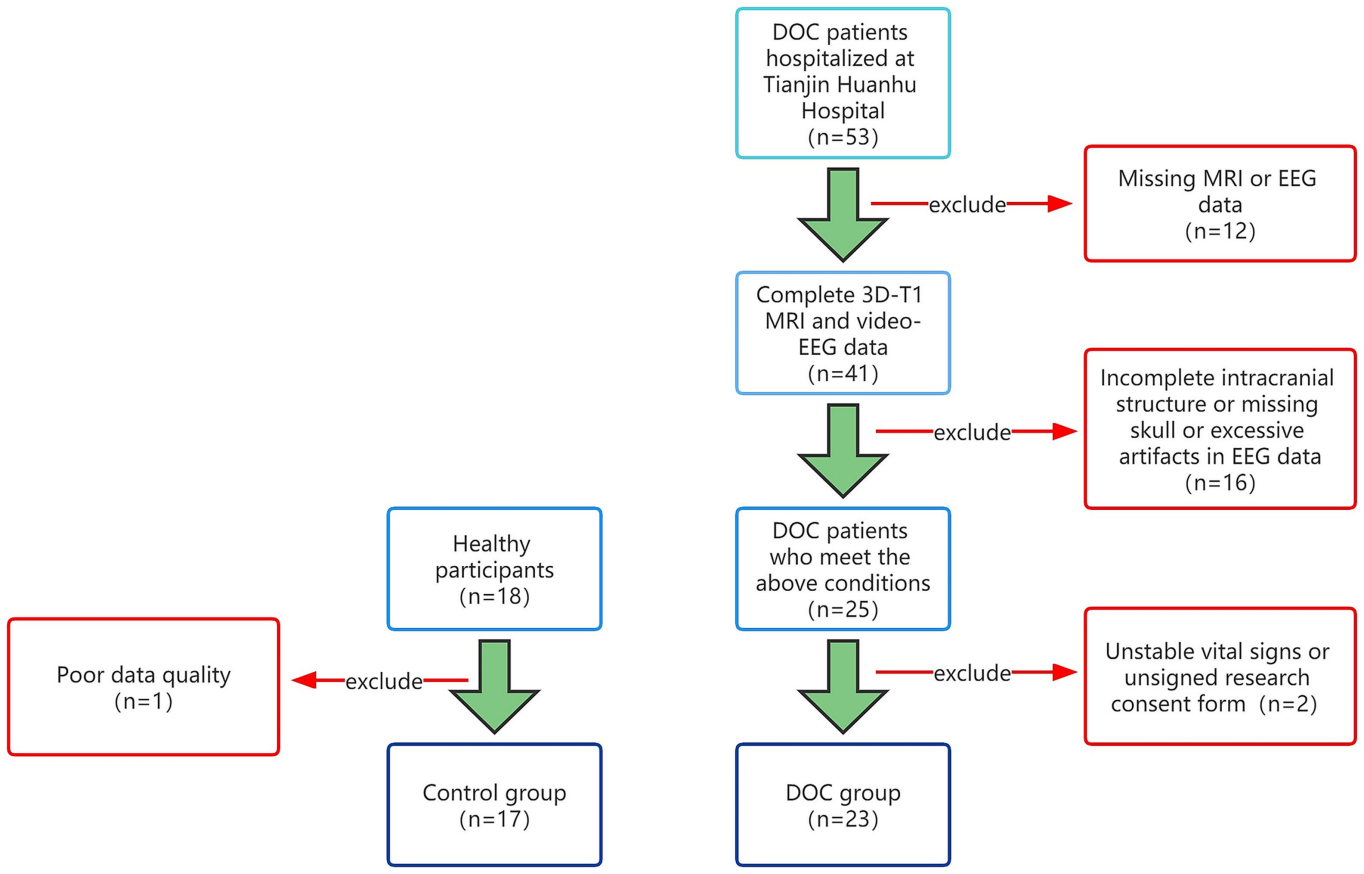
Group flowchart.

This study included pDOC patients who were treated and evaluated in the Neurosurgery Department from January 2021 to January 2023. Inclusion criteria are as follows: 1. Disorders of consciousness greater than 28 days; 2. After admission, a professional physician should complete at least 3 CRS-R scores evaluations; 3. Video EEG monitoring is more than 12 h; 4. Improve head MRI examination; 5. The intracranial structure is relatively intact, without skull defects; 6. The clinical data is complete. The exclusion criteria are as follows: 1. Poor data quality and high artifacts; 2. Application of sedative and anesthetic drugs during data collection; 3. Unstable vital signs. This study was approved by the Medical Ethics Committee of Tianjin Huanhu Hospital Hospital and obtained the written informed consent of all participants or legal guardians. Then, collected the resting-state EEG and head MRI data of 17 healthy participants. This study was divided into two groups based on the presence or absence of pDOC, consisting of 17 healthy individuals and 23 pDOC individuals (Figure 1).

### Data acquisition

Collect general information on pDOC patients, including age, gender, etiology, CRS-R score before EEG monitoring, and CRS-R score at 2-month follow-up. Collect age and gender data for the HG. The MRI sequence was scanned using SIEMENS SKYRA 3 T magnetic resonance imaging, and a T1 weighted three-dimensional magnetization prepared rapid-gradient echo imaging (3D-T1-MPRAGE) was collected, with TR = 2000 ms, TE = 3.0 ms, and layer thickness = 1 mm. Flip angle = 9°, Slice Gap: 50 percent, Resolution Matrix = 256 × 256. The MRI scanning lasts for 4 min and 40 s. The video EEG signals were sampled with the Nuclecle Medical Evoked Potential Instrument. The electrode position was placed according to the international 10–20 system, with a sampling rate 2 kHz and a band-pass filter of 0.1 Hz-250 Hz, and the impedance of all recording electrodes was kept below 10 k *Ω*. EEG data were recorded with Medical Evoked Potential Instrument Software (Nutricle, China). Each healthy participant is required to open their eyes and rest while collecting data in a quiet room, with their thoughts cleared and not falling asleep. The EEG selected by each pDOC subject is the EEG during the patient’s eye-opening.

### Microstate analysis

To analyze EEG data, it is necessary to preprocess to obtain a section of clean EEG data. We use the toolbox EEGLAB 2023.0 based on Matlab2018b (Mathworks Inc., Natick, Massachusetts, United States) for preprocessing. Firstly, import data and locate channels. We use standard international 10–20 system electrodes and then filter the data with bandpass filtering of 1-30 Hz. Due to substantial artifacts in the EEG data of pDOC patients, we focused on the 1–30 Hz frequency band for analysis. Remove the baseline and remove bad channels. EEG is a mixture of source signal and noise and eliminates artifacts by Independent Component Analysis (ICA) to form an EEG source signal. Remove components from topographic maps obtained through ICA. Then, reject extreme values. Finally, perform a re-reference using the whole brain average reference.

**Figure 2:**
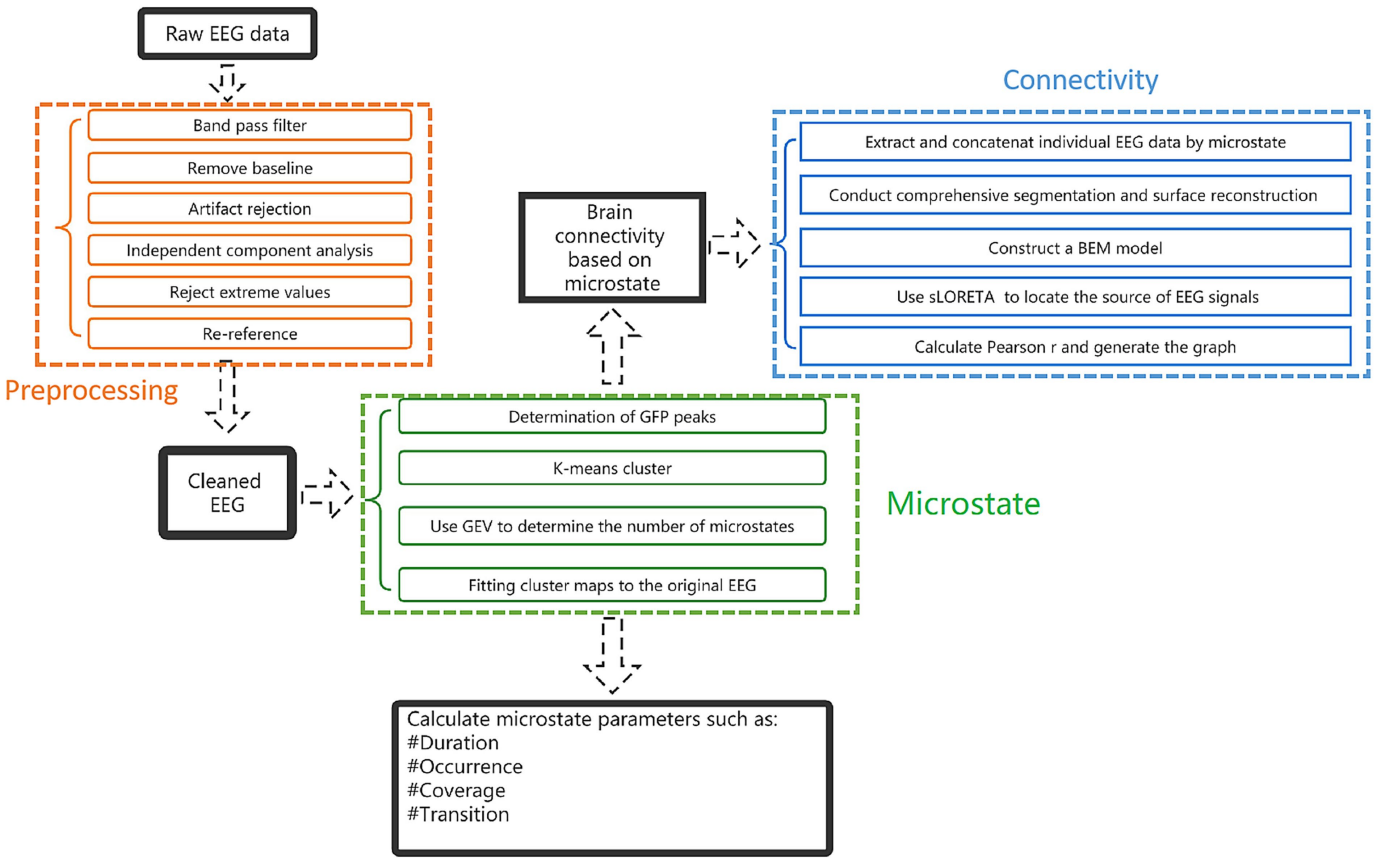
Research step flowchart.

Microstate analysis toolbox (v1.0) in EEGLAB was used for microstate analysis. Firstly, clustering is carried out, clustering was calculated by a polarity-insensitive modified k-means algorithm. The K range is set from 2 to 8, with a random start value, and repeated 50 times. Pascual et al. proposed a statistical approach that directly considers the topology of the whole map, rather than reducing it to the position of the extreme. This method based on K-means clustering analysis grouped the maps with high spatial correlation in the way of nested iterative fashion and determines the representative terrain that can best explain the variance in each cluster (41). These topographic maps regardless of polarity. The number of prototype plots is determined by the Global Explained Variance (GEV) in the range of K and the optimized value of cross validation criteria. Once the cluster plots were determined, they were fitted to the individual EEG data to define the MS, extract different time parameters of each participant, and compare these parameters between experimental conditions or between participant groups. After obtaining the topographic map, the following parameters are calculated and counted: (1) Duration: the average duration for a given MS to remain stable. (2) Occurrence: frequency of a MS in an individual. (3) Coverage: the percentage of a MS in the total recording time. (4) The global variance explained by each MS (41) (Figure 2).

### Brain connectivity

EEG connectivity metrics were measured using the toolbox Brainstorm^1^. The EEG data were reconstructed according to the global field power (GFP) of clustered MS, and the connectivity of the four MS was calculated, respectively. The previous step of EEG pre-processing has been completed. After re-reference, the participant’s MRI 3D-T1 sequence was imported, and the EEG electrode was positioned to the MRI image. FreeSurfer^2^ was used to perform a comprehensive segmentation and surface reconstruction of structural MRI, forming a high-definition cortical layer and the boundary surface of the brain, skull, and scalp. These surfaces were then used to construct a boundary element method (BEM) model. Conductivity values were assigned to each interval. The standard contour of the electrode position was digitized and co-registered with the reference point on the template brain. The high-density cortical mesh is used as the source space. Then, the standardized low resolution brain electromagnetic tomography (sLORETA) is used to locate the source of EEG signals. The brain was divided according to Desikan-killiany Division (42). Finally, calculated Pearson *r* and generated the connectivity circos map (Figure 2).

### Statistical analysis

All statistical calculations were performed in SPSS 26.0. Metrological Data in line with normal distribution are expressed as mean ± SD and categorical variables are reported as numbers (*n*) and proportions (%). A nonparametric test was used to compare the components of non-normal distribution measurement data. *p* < 0.05 was considered statistically significant.

## Results

### Participant characteristics

There were 17 participants in HG with an average age of 29.2 ± 8.3 years, including 10 (58.8%) males and 7 (41.2%) females. There were 23 patients in DG with an average age of 42.52 ± 11.89 years, including 16 (69.6%) males and 7 (30.4%) females. The etiology was cerebral hemorrhage in 17 (74%), brain injury in 3 (13.0%), and hypoxic–ischemic encephalopathy in 3 (13.0%). Prior to data collection, based on the CRS-R assessments, 13 patients (56.5%) were classified as being in a Vegetative State (VS), 9 (39.1%) were in a Minimally Conscious State minus (MCS-), and 1 (4.4%) was in a Minimally Conscious State plus (MCS+). At follow-up, 8 patients (34.8%) showed an improvement in their CRS-R classification (Supplementary Tables).

### Microstate analysis

**Figure 3:**
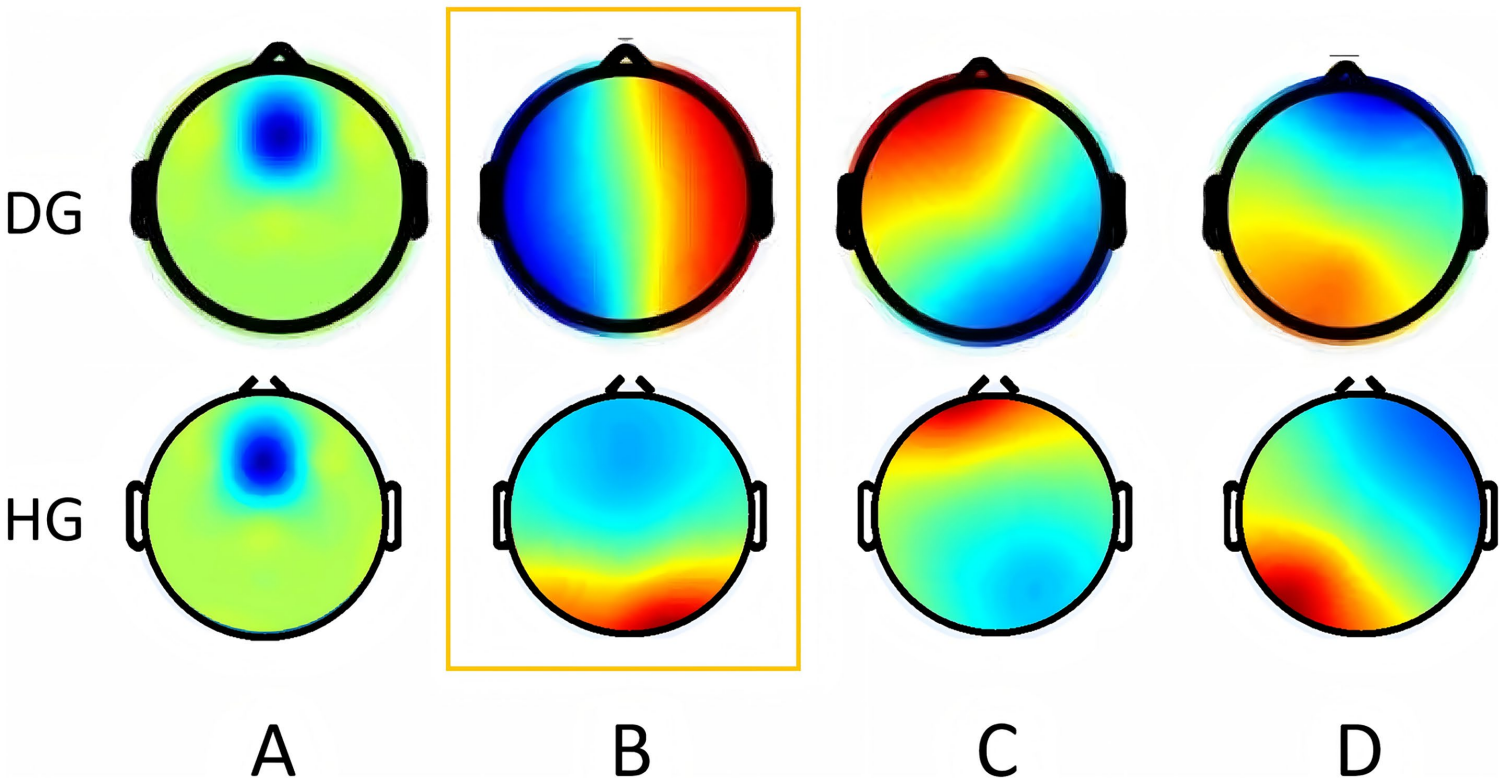
Microstate topographic map. The upper row shows the topographic map of the DOC group. The bottom row shows the topographic map of the health group. Orange boxes represent differential topographic maps. DG, DOC group; HG, Health group. (The colors used in the topography contours are arbitrary and do not carry any specific meaning).

After clustering analysis, four MS were obtained for each of the two groups. They are named A, B, C, and D (Figure 3). Among them, the topography of MS A, B, and D groups is similar, while MS B has significant differences in morphology. MS A is a front-back distribution, with the maximum value at the forehead. MS B is distributed anteriorly and posteriorly in the HG, with the maximum value in the occipital region, but in the DG, MS B is distributed left–right. The MS C and D are similar in both groups, with left frontotemporal-right parietal occipital and right frontotemporal-left parietal occipital directions. The average global explanatory variance (GEV) of the DG was 0.73 ± 0.95, while the average GEV of the HG was 0.70 ± 0.51.

**Figure 4:**
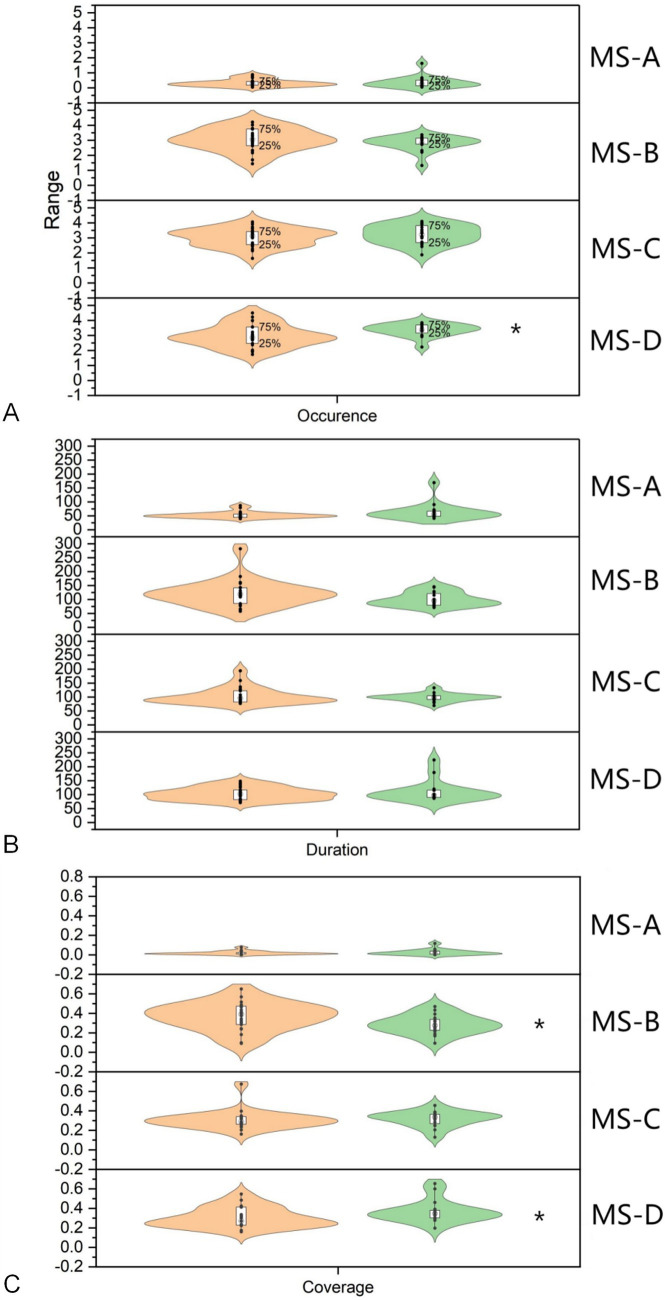
Statistical analysis of microstate indicators. **(A)** The Occurrence of microstate D in the DOC group was 2.96 ± 0.75, the value in the healthy group was 3.36 ± 0.42, indicating a significant difference (*Z* = −2.141, *p* = 0.032). **(B)** The Coverage of microstate B in the DOC group was 0.37 ± 0.14, the value in the healthy group was 0.29 ± 0.10, indicating a significant difference (*Z* = −2.084, *p* = 0.037). **(C)** The Coverage of microstate D in the DOC group was 0.30 ± 0.10, the value in the healthy group was 0.37 ± 0.12, indicating a significant difference (*Z* = −1.999, *p* = 0.046). * *p*<0.05 HG, Health group; DG, DOC group.

Among the indicators of MS A, the Occurrence in the DG was 0.33 ± 0.24, while the value in the HG was 0.38 ± 0.39, with no statistical significance (*Z* = −0.114, *p* = 0.921). The Duration of DG was 53.11 ± 11.39, while the value of the HG was 63.95 ± 30.82, which was not statistically significant (*Z* = −1.370, *p* = 0.177). The Coverage of the DG was 0.02 ± 0.02, while the value for the HG was 0.03 ± 0.03, which was not statistically significant (*Z* = −0.485, *p* = 0.641). Among the indicators of MS B, the Occurrence in the DG was 3.04 ± 0.74, while the value in the HG was 2.79 ± 0.50, with no statistical significance (*Z* = −1.71, *p* = 0.251). The Duration of DG was 121.53 ± 47.29, while the value of the HG was 100.60 ± 24.56, which was not statistically significant (*Z* = −1.456, *p* = 0.151). The Coverage of DG was 0.37 ± 0.14, while the value of the HG was 0.29 ± 0.10, which was statistically significant (*Z* = −2.084, *p* = 0.037). About the MS C, the Occurrence in the DG was 3.02 ± 0.61, while the value in the HG was 3.21 ± 0.65, with no statistical significance (*Z* = −0.885, *p* = 0.388). The Duration of DG was 103.30 ± 29.32, while the value of the HG was 98.57 ± 14.61, which was not statistically significant (*Z* = −0.542, *p* = 0.601). The Coverage of the DG was 0.31 ± 0.97, while the Coverage of the HG was 0.32 ± 0.08, which was not statistically significant (*Z* = −1.313, *p* = 0.196). In MS D, the Occurrence in the DG was 2.96 ± 0.75, while the value in the HG was 3.36 ± 0.42, which was statistically significant (*Z* = −2.141, *p* = 0.032). The value of Duration in the DG was 102.98 ± 22.50, while the value in the HG was 111.18 ± 37.83, which was not statistically significant (*Z* = −0.314, *p* = 0.767). The Coverage of the DG was 0.30 ± 0.10, while the Coverage of the HG was 0.37 ± 0.12, which was statistically significant (*Z* = −1.999, *p* = 0.046). Figure 4 shows the comparison of parametric statistics of four MS.

### Connectivity

**Figure 5:**
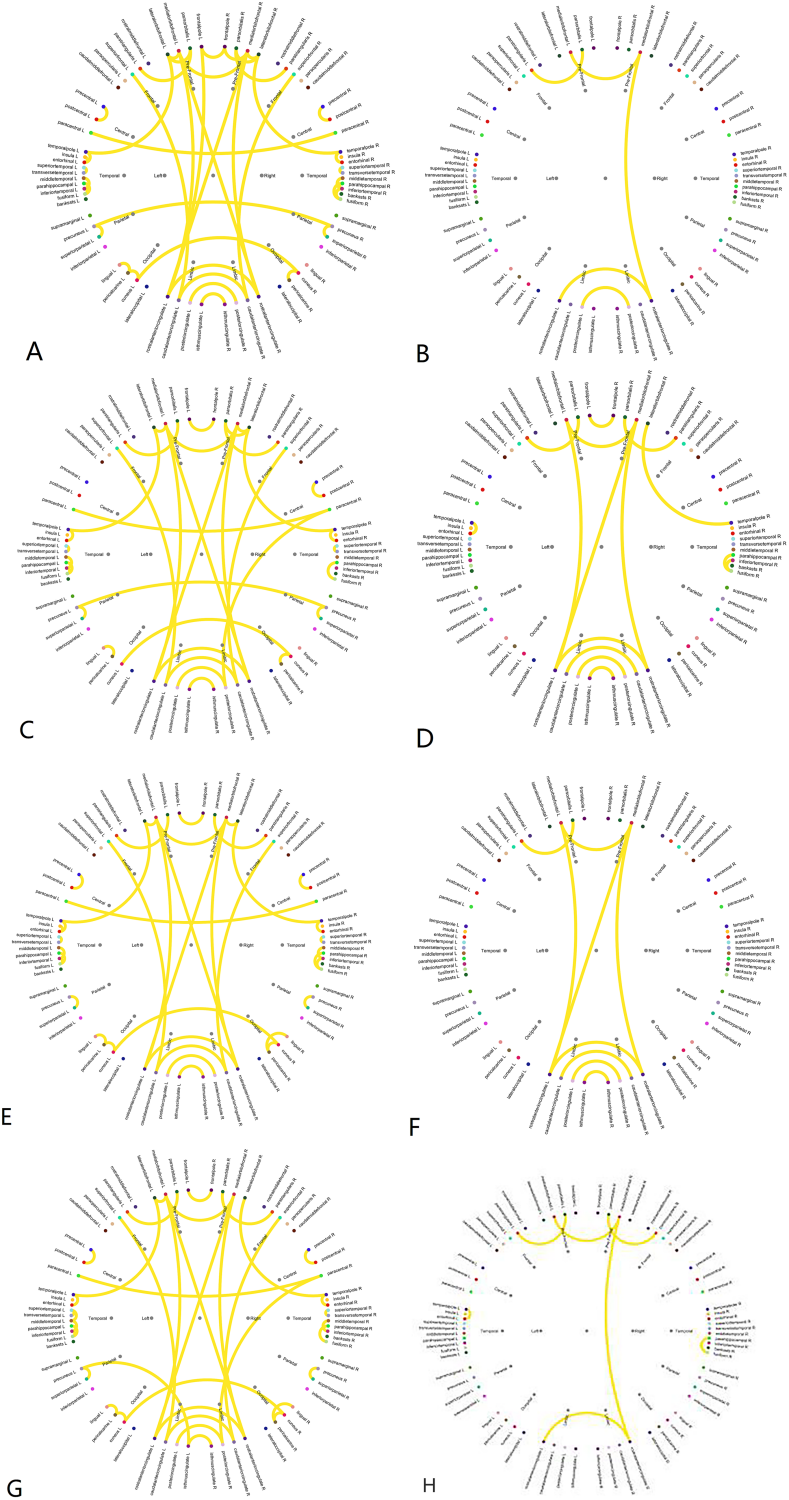
Pearson correlation coefficient brain connectivity map. **(A)** Brain connectivities of microstate A in the healthy group. **(B)** Brain connectivities of microstate A in the DOC group. **(C)** Brain connectivities of microstate B in the healthy group. **(D)** Brain connectivities of microstate B in the DOC group. **(E)** Brain connectivities of microstate C in the healthy group. **(F)** Brain connectivities of microstate C in the DOC group. **(G)** Brain connectivities of microstate D in the healthy group. **(H)** Brain connectivities of microstate D in the DOC group.

The brain connectivity circos maps of each MS between the two groups are shown in (Figure 5). Regardless of the MS, under the same threshold, the connectivity of the DG was significantly lower than that of the HG, especially with a significant decrease in connectivity between brain regions. In MS A, only part of the connections between frontal lobes, part of the prefrontal lobe to the limbic system, and a small part of connections within the limbic system are retained in DG, and there is almost no connection between hemispheres. The MS B topographic map was significantly different between the two groups. Compared with the HG, the MS B of DG lost a lot of connections from the frontal lobe to the temporal lobe and the limbic lobe, and the connections between the bilateral central areas were reduced. In MS C, the main difference between the two groups was a decrease in connectivity between the temporal lobes, bilateral central regions, and bilateral occipital lobes in the DG. The MS D of DG was significantly different from that of the HG. Only part of the frontal lobe, a small amount of temporal lobe connection, a small amount of limbic lobe connection, and the connection between the left prefrontal lobe and the limbic lobe were preserved. Analysis of the connectivity of the four microstates using a *Z*-test (Table 1) revealed significant differences, with the CG exhibiting significantly higher connectivity than the DG in all states (Figure 5).

**Table 1:** The results of the *Z*-test for connectivity.

Microstate	CG	DG	*Z*	*p*
MS A	0.76 ± 0.11	0.69 ± 0.14	−21.39	<0.01
MS B	0.82 ± 0.08	0.74 ± 0.12	−25.77	<0.01
MS C	0.83 ± 0.08	0.70 ± 0.13	−35.15	<0.01
MS D	0.83 ± 0.08	0.69 ± 0.13	−34.62	<0.01

## Discussion

MS analysis is the analysis of brain activity from a time series. This method first identifies several microstates at the group level and then projects them onto individual participants, thereby studying the different combinations and arrangements of MS in each participant. The transition between MS can be explained as the sequential activation of different neural networks, while the time series of MS in resting-state EEG can allow us to visually observe the fast switching between the collective activities of neurons at rest. There are three main indicators of MS, *Duration* refers to the average length of time a specific MS remains stable when it appears, *Coverage* is the proportion of the microstate to the total recording time, and *Occurrence* is the average number of times per second that the MS dominates during the recording period (28). This study analyzed the MS of pDOC patients and found that the morphology of MS B is unique to pDOC and can be used as a topographic map to distinguish between healthy and pDOC groups.

Studies have shown that the four MS have a spatial correlation with fMRI. MS D is mainly related to BOLD signal changes in the bilateral superior temporal gyrus and is considered to be related to language processing (43). This study shows that the Occurrence and Coverage of MS D in the DG are significantly lower than those in the HG. Therefore, pDOC patients may have obstacles in understanding instructions and are unable to communicate effectively with the outside world. From the perspective of connectivity, compared to other MS, MS D has a more significant decrease in connectivity compared to the HG. Therefore, the impairment of language comprehension function in pDOC patients may be an important reason for communication barriers with the outside world. Recent studies by Stefan et al. have also shown that the MS from the lower left and upper right is very effective in predicting coma outcomes, especially in *Duration* and *Occurrence* in the delta frequency band (0-4 Hz) and theta frequency band (4-8 Hz) (43). MS C is believed to be related to visual function (44). MS B is related to the activities of the anterior cingulate cortex, inferior frontal gyrus, claustrum, and frontal insular cortex (44). This region is closely related to the “Salience Network (SN),” which plays a switching role between the central executive network and the DMN. SN is the “mediator” of the brain, which will continuously monitor the external world and carefully determine the response of other brain networks to new information and stimuli (45). Highlighting the structural and functional integrity of the network is necessary to regulate the activity of the DMN. The MS B of pDOC group can be used as the characteristic topographic map of pDOC, and the Duration, Coverage and Occurrence of microstate in this topographic map are increased than those in the healthy group, and the Coverage is also significantly increased. Therefore, MS B of the pDOC group is of great significance both in the morphology of the topographic map and in the statistics of various micro state indicators, that is, the prominent network of pDOC is significantly damaged. MS A is associated with BOLD signals in the frontal and parietal cortex and is believed to be related to attention (44). The combination of MS and fMRI also indicates that the brain network depicted by fMRI is the sequential activation of different network components in time, rather than the simultaneous activity of all network components. The reason for this change is the low temporal resolution of MRI (46), and MS based on high temporal resolution EEG precisely compensates for this deficiency. The different combinations of various MS in time series also indicate that the brain is active every moment. Recent studies have pointed out through training a random forests classifier that MS may be the electrophysiological basis for brain dynamic functional connectivity (47).

Functional connectivity measures the statistical dependence of physiological time series recorded in different regions of the brain. Since the calculation of functional connectivity highly depends on brain activity changes in time series, high temporal resolution techniques such as EEG (<1 ms) are the best tool to reflect neural dynamics. We found that the four MS were significantly different between pDOC and healthy groups, that is, in the 1-30 Hz EEG time series, the brain connectivity of pDOC patients was significantly reduced. These results are similar to those of Lechinger et al., and the level of connectivity is related to the severity of pDOC (48). A study on neurocognitive dysfunction and pDOC showed that in the DG, EEG connections in all frequency bands were reduced, and there were also significant differences in connections from other cortical regions to frontal lobe regions (49), which seemed to indicate the role of the frontal lobe in consciousness. In this study, the connections related to the frontal lobe, especially the connections from the frontal lobe to the limbic lobe, were significantly reduced. We also found that the directional connection between the hemispheres of pDOC is reduced, and the information communication seen in the cerebral hemisphere seems to be closely related to the level of consciousness, which needs further research to prove.

The study by Xu et al. revealed that patients exhibiting microstate B, which is also present in healthy individuals, tend to have a more favorable prognosis (50). The parametric characteristics of microstate B demonstrated a strong correlation with clinical scale scores, thereby establishing it as the most accurate indicator reflecting the level of consciousness. This finding suggests a close association between microstate D and the level of consciousness (50). In a study involving pediatric pDOC patients, the results demonstrated a significant correlation between microstate-related parameters and CRS-R scores, suggesting that microstates have the potential to predict consciousness recovery (51). According to Liuzzi et al., microstates may be necessary to sustain consciousness, but cannot be considered as the only actor in consciousness presence/recovery, they also contend that the emergence of consciousness is dependent on the existence of an anterior–posterior topography (Microstate B), a map closely linked to frontoparietal activity, aligning with our study (52).

There are also some shortcomings in this study. This study included a small data volume, especially for patients in the DG, so pDOC was not further classified based on CRS-R scores or etiology. Although there was a significant age difference between the two groups, no further age-based stratification was performed in this study, given that microstate topographies and related metrics exhibit only subtle variations among adults, except in children and elderly individuals. This may have introduced some bias into the results. In the analysis, we found that although the DG had three MS topographical maps similar to the HG, the underlying neurological mechanisms may not be the same. For example, pDOC patients often have pathological changes such as cortical atrophy and subcortical fiber bundle destruction, which are inevitably accompanied by neurological dysfunction and changes in brain networks. The brain connectivity analysis of this study also showed significant differences between the two groups. Therefore, exploring the neuro-electrophysiological mechanisms of the abnormal brain is the focus of our next research.

## Conclusion

The MS B of pDOC patients is unique compared to the HG and may be a biomarker for distinguishing pDOC from healthy individuals. At the same time, the brain connectivity of pDOC patients in the four MS decreased significantly compared to the HG, especially the connectivity between brain regions and hemispheres. Finally, microstate analysis combined with connectivity analysis showed that Significant Network damage was the key to affect consciousness.

## Data Availability

The original contributions presented in the study are included in the article/Supplementary material, further inquiries can be directed to the corresponding author.

## Footnotes

^1^ https://neuroimage.usc.edu/brainstorm

^2^ https://surfer.nmr.mgh.harvard.edu
