# Supplementary material for: Analysis of the microstates of prolonged disorders of consciousness
Source: Front Neurol. 2025 Oct 29;16:1659809. doi: 10.3389/fneur.2025.1659809 (PMC12605022; doi:10.3389/fneur.2025.1659809)
Supplement: Supplementary file 1 [file Table_1.docx]

| Number | Sex | Age(y) | Etiology | CRS-R# | Follow-up CRS-R |
| --- | --- | --- | --- | --- | --- |
| 1 | Female | 64 | HS | VS | MCS- |
| 2 | Male | 49 | HS | MCS- | MCS- |
| 3 | Male | 55 | HS | VS | VS |
| 4 | Male | 44 | HS | MCS- | MCS+ |
| 5 | Female | 40 | HIE | VS | VS |
| 6 | Female | 36 | HS | VS | MCS+ |
| 7 | Male | 36 | TBI | VS | MCS- |
| 8 | Male | 40 | HS | MCS- | MCS- |
| 9 | Female | 19 | TBI | VS | MCS+ |
| 10 | Male | 50 | HS | MCS- | MCS- |
| 11 | Female | 59 | HS | VS | VS |
| 12 | Male | 51 | HS | MCS- | MCS- |
| 13 | Male | 43 | HS | VS | VS |
| 14 | Male | 32 | HS | VS | VS |
| 15 | Male | 50 | HS | VS | VS |
| 16 | Male | 51 | HIE | MCS- | MCS- |
| 17 | Male | 51 | HS | VS | MCS- |
| 18 | Male | 38 | HS | MCS- | MCS+ |
| 19 | Female | 32 | TBI | VS | eMCS |
| 20 | Male | 38 | HS | MCS+ | MCS+ |
| 21 | Male | 15 | HIE | VS | VS |
| 22 | Male | 33 | HS | MCS- | MCS- |
| 23 | Female | 52 | HS | MCS- | eMCS |

**Supplemental Table** Characteristics of the DOC group (n=23). #: CRSR score evaluated during Electroencephalography. HS: Hemorrhagic stroke HIE: Hypoxic-ischemic encephalopathy TBI: Traumatic brain injury VS: Vegetative state MCS: Minimally conscious state eMCS: escape MCS

| Name | Age(y) | Sex |
| --- | --- | --- |
| CW | 20 | F |
| CYF | 38 | M |
| DYL | 20 | M |
| FKK | 37 | M |
| HYM | 20 | F |
| JZP | 20 | F |
| LJC | 35 | M |
| LJL | 38 | F |
| LZZ | 35 | M |
| SY | 40 | M |
| WHL | 38 | F |
| WYZ | 30 | M |
| WZT | 35 | M |
| XPC | 30 | M |
| YG | 18 | M |
| ZJ | 22 | F |
| ZY | 20 | F |

**Supplemental Table** Characteristics of the Healthy group (n=17). #:F:Female M:Male
